# Uncovering the network structure of non-centrally cleared derivative markets: evidence from large regulatory data

**DOI:** 10.1007/s00181-023-02396-9

**Published:** 2023-03-10

**Authors:** Sebastiano Michele Zema

**Affiliations:** 1 European Central Bank, Frankfurt am Main, Germany; 2 Institute of Economics, Sant’Anna School of Advanced Studies, Pisa, Italy

**Keywords:** Complex networks, Financial derivatives, Non-centrally cleared exposures, Maximum spanning trees, G01, G15, G23

## Abstract

The network structure of non-centrally cleared derivative markets, uncovered via the European Market Infrastructure Regulation, is investigated by reconstructing initial and variation margin networks to analyze channels of potential losses and liquidity dynamics. Despite the absence of central clearing, the derivative network is found to be ultra-small and a maximization-based filtering tool is proposed to identify channels in the network characterized by the highest exposures. I find these exposures to be mainly toward institutions outside the euro area, emphasizing the need for cooperation across different jurisdictions. Anomalous behavior in terms of diverging first and second moments of the degree and strength distributions are detected, signaling the presence of large exposures generating extreme liquidity outflows. A reference table of parameters’ estimates based on real data is provided for different network sizes, with no break of confidentiality, making it possible to simulate in a realistic way the liquidity dynamic in global derivative markets even when access to supervisory data is not granted.

## Introduction

The recent default of Archegos Capital Management in March 2021, caused by exposures on Total Return Swaps (TRS) on equities and combined with the adoption of poor margining practices, signaled how appropriate risk mitigation techniques and supervision must take place when derivative contracts are not centrally cleared by central clearing counterparties (CCPs). According to the latest statistical release from the Bank of International Settlements (BIS) on OTC derivatives statistics, central clearing rates for interest rate derivatives (IRDs) and credit default swaps (CDS) stand, respectively, at 78% and 62% of total notional at the end 2021^1^. Moreover, some types of equity derivative instruments as Contract for Differences (CFD) and Total Return Swaps (TRS) are mostly exchanged bilaterally among counterparties with no central clearing in place^2^.

This paper sheds light on bilateral derivative transactions that have not been centrally cleared through CCPs by using the voluminous transaction-by-transaction data collected under the European Market Infrastructure Regulation (‘EMIR data’). The collateral network structure of non-centrally cleared derivative markets is uncovered, analyzing the implications of the detected structures from a systemic risk and monitoring perspective. The implementation of a filtering tool to extract relevant information from such large and complex networks will be proposed, also providing the literature with some reference for the parameters’ estimates to promote the adoption of appropriate distributional assumptions for the modeling of such networks. This will allow other researchers, with no access to this kind of confidential data, to simulate and replicate the structure of derivative markets in a more realistic way.

In the aftermath of the 2008 Global Financial Crisis, the weaknesses of derivatives markets and the necessity to increase their transparency become evident to the regulators. Despite the important role played by financial derivatives in the economy, these instruments embed substantial risks which were significant drivers of the collapse of Lehman Brothers and the near-failure of AIG (McDonald and Paulson 2015). To this end, the G20 leaders decided at the 2009 summit in Pittsburg to make over-the-counter (OTC) derivatives trading safer and more transparent.

In Europe, to implement these goals, the initiative was formalized in 2012 in the European Markets Infrastructure Regulation (EMIR) which requires EU entities engaged in derivative transactions to report them to trade repositories (TRs) authorized by the European Securities Markets Authority (ESMA). To reduce counterparty credit risk, a clearing obligation through central clearing counterparties (CCPs) for standardized derivative products and stricter margining practices were introduced. Under the current EMIR technical standards, precise clearing thresholds are set by the class of OTC derivative contracts, which determine whether Financial counterparties (FC) and Non-Financial counterparties (NFC) are subject to clearing obligations through CCPs. Whenever a derivative trade is not centrally cleared, risk mitigation techniques must take place and the regulation requires counterparties to exchange variation margins (VM) and initial margins (IM).

Both IM and VM are used as collateralization instruments and will be used in this paper to uncover the network structure of non-centrally cleared derivative exposures. The IM is posted at the beginning to enter a derivative contract and it is intended to cover potential losses arising in the period between the defaulter’s last variation margin payment and the point in time at which the surviving party is able to hedge or replace the trade. The higher the IM posted the higher the wealth at stake in that bilateral transaction. The amount of IM can be recalibrated over time, especially in a period of high market volatility (i.e., re-coverage of portfolios’ riskiness). The VM is used to compensate for daily fluctuations in the market value of the derivative contract instead. Given a certain variation in the price of the underlying instrument, the counterparty negatively affected by the price variation is required to pay a certain amount of money accordingly. Then, the IM network will reflect the riskiness in terms of potential future losses of the derivative exposures while the VM network constitutes, for a given time span, the liquidity flows of money between the involved institutions.

Two main findings will be highlighted in the paper. First, the channels containing the highest exposures, and identified through the proposed filtering tool, involve mainly institutions belonging to jurisdictions outside the euro area (EA). Second, both initial and variation margin networks display anomalous behaviors in the power-law parameter associated, respectively, with both the degree and in/out-strength distributions, signaling the presence of extreme positions and liquidity outflows during turmoil periods to be brought to the attention of policymakers and supervisory authorities.

The article is organized as follows: In Sect. 2, the literature on empirical and network studies that contributed to this strand of research on financial derivatives is briefly reviewed; in Sect. 3, the data set is described; in Sect. 4, both the IM and VM networks are analyzed, emphasizing their structural properties and subsequent potential implications; conclusion and discussions are finally provided in Sect. 5.

## Literature review

The literature on the network structure of derivative markets is particularly small due to lack of access to confidential and regulatory data. On the empirical side, given the 2008 financial crisis and subsequent sovereign debt crises, the literature started to implement network approaches to monitor and assess the systemic risk arising from Credit Default Swaps (CDS) markets and potential contagion channels through highly interconnected institutions (Markose et al. 2012; Cetina et al. 2018). These studies confirmed the importance of monitoring CDS exposures given their level of concentration in the market and the presence of potential ‘super-spreaders’ of contagion. Duffie et al. (2015) also used bilateral CDS positions to estimate the impact of clearing and margining practices on collateral demand. A detailed analysis on the network structure of the CDS markets can be found also in Peltonen et al. (2014), where the economic determinants of the network and its emerging properties were econometrically investigated using a relatively large sample of 642 entities transacting in the CDS market.

Another strand of literature focused instead on CCPs stability, investigating the riskiness of their clearing members positions and the effectiveness of the client-clearing intermediation process. Despite the general agreement on the crucial role played by CCPs in reducing counterparty credit risk in derivative transactions, Duffie and Zhu (2011) provided an interesting assessment for the role of CCPs and critically analyzed the impact of central clearing on counterparty risk. Concentrating risks in CCPs and their implications for network stability in OTC derivative markets have been widely investigated also by Heath et al. (2016), where data on margins were simulated, given the lack of access to regulatory data, using aggregate balance sheet data and historical time series prices to calibrate the simulations.^3^

The network structure of OTC derivative markets in the UK has been examined also by Bardoscia et al. (2019) who highlighted the usefulness of network centrality measures as proxies for the vulnerability of clearing members, and the potential liquidity risk they may face because of the directionality of their portfolios. The liquidity risk faced by clearing members under different stress scenarios has been investigated also recently by Bardoscia et al. (2021) who used trade repository data for a sample of 103 clearing members.

With respect to the current literature, this contribution is manifold. First, I shed light on that part of the derivative market that is not subject to central clearing and which has not been sufficiently covered in the literature. I thus provide insights into the implications of the detected network structures from a financial stability point of view. Second, differently from previous studies, I include in the sample all the transacting entities reporting to the EMIR infrastructure. Moreover, to have a complete view of the overall exposure for each reporting institution, I collect initial and variation margin data across all the different kinds of derivative instruments (mainly Swaps, Futures/Forward and Options) and underlying asset classes (mainly Interest Rates, Credit, Equities and Commodities). This leads to a very large network. I thus propose the adoption of a filtering tool to extract relevant information from the initial margin network, selecting a subset of exposures that might generate large losses for counterparties. Finally, I contribute to the literature by providing a reference table containing estimates and non-confidential topological information on the variation margin network. This might enable researchers to simulate derivative networks in a more realistic way, to study liquidity risk and contagion, even with no access to regulatory data.

## Data description

EMIR requires counterparties to report their derivative transactions to the TRs. All derivative transactions, both centrally cleared and bilateral contracts, should be reported independently of whether traded on exchange-traded derivative (ETD) markets or on OTC markets. The regulation applies to financial intermediaries as well as to non-financial companies with positions above some thresholds defined by the regulation. It is a double-reporting system, meaning the reporting obligation is on both counterparties. The TRs collect and maintain the records of the derivative contracts reported and make them available to the relevant authorities. The European Central Bank (ECB) is entitled to receive all the transactions in which at least one of the following situations holds true: (i) At least one counterparty in the derivative transaction is resident in the euro area (i.e., a future contract between two counterparties being, respectively, in Germany and UK); (ii) the reference entity is in the euro area (a CDS on a German bank, even if transacted between extra euro area institutions); and (iii) the reference obligation is the sovereign debt of a euro area country (i.e., a CDS on Italian debt).

The data set is highly granular. For each trade on a derivative instrument, in which at least one of the conditions above-mentioned is met, a huge amount of information covering details on both the derivative instrument, the underlying, the exact time at which the trade occurred, and the two counterparties involved is available. Since a set of multiple contracts can be traded between two generic counterparties, the net position resulting from these multiple contracts is also reported in EMIR. This net position constitutes a portfolio. All the trades constituting a portfolio are assigned a unique code, which makes it possible to understand which portfolio that specific trade belongs to. The amount of initial and variation margin exchanged and reported by two generic counterparties reflects the overall riskiness of their multiple contracts. Even if each single trade is separately recorded in the data set, the fields containing the amount of collateral posted and received do not refer to the trade standalone but to the entire portfolio that trade belongs to. Thus, to reconstruct both the initial and variation margin networks, all the portfolios have been at first identified through the portfolio codes reported in a trade record.

After having reconstructed all the portfolios for all the reporting counterparties, the associated initial and variation margins associated with those portfolios have been sampled and used to reconstruct the IMs and VMs collateral networks at the micro (counterparty) level.^4^ The portfolios on which IMs and VMs are calibrated can include either different types of derivative instruments, different types of underlying in terms of asset class, or even both different asset classes and contract types at the same time. I will refer to portfolios that contain either one type of derivative instrument only (i.e., only futures) or one type of underlying only (i.e., derivatives on interest rates only for instance) as ‘pure portfolios’ with respect to the dimension of interest.

**Fig. 1:**
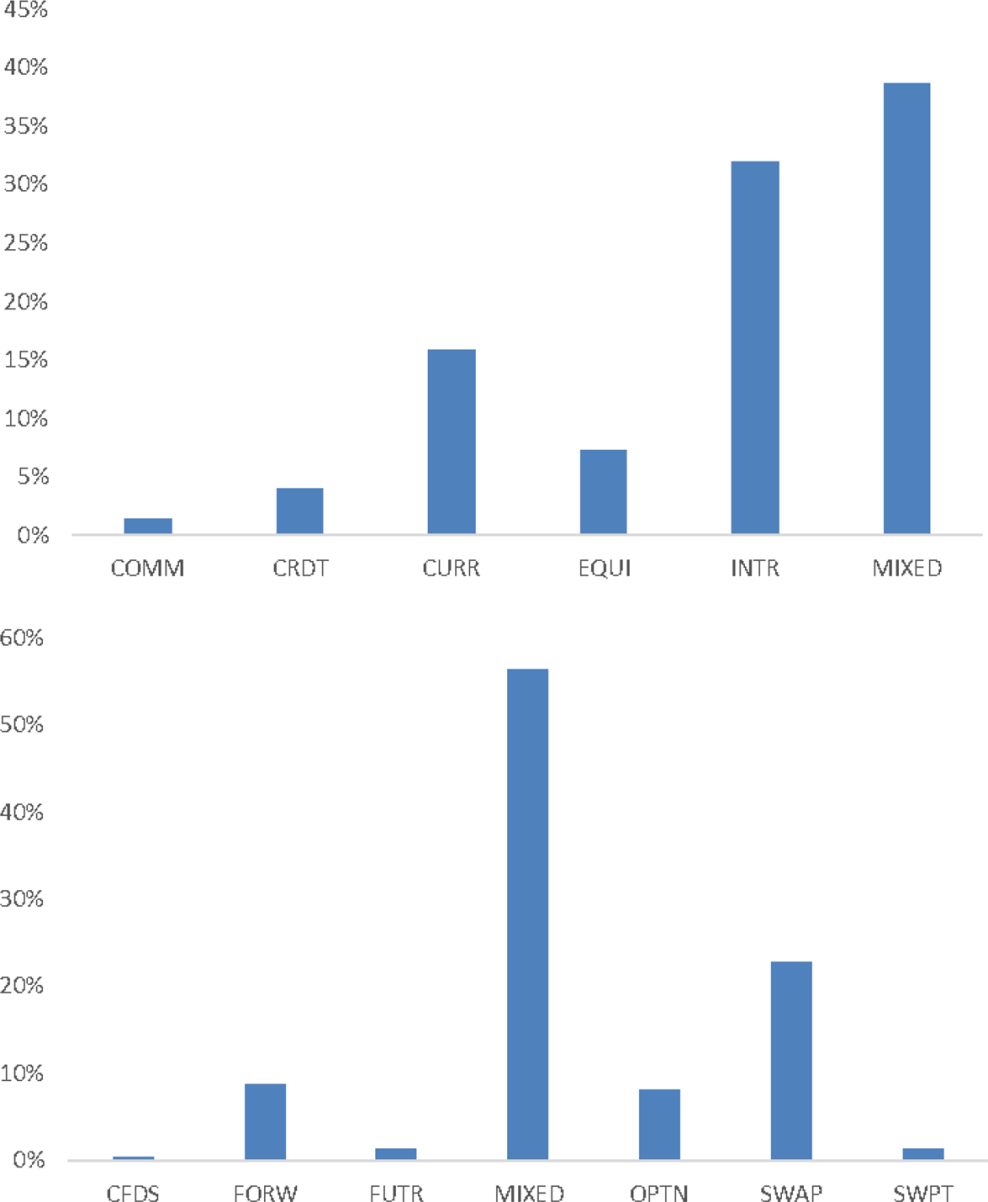
Almost 60% of the total notional size can be attributed to portfolios containing multiple derivative instruments. Almost 40% of the same notional can be attributed to portfolios containing multiple asset classes as underlying. The portfolios being mixed both in terms of derivative instruments and asset classes represent 36% of the overall notional. For ‘pure’ portfolios, Swaps contracts (23%) are the mostly used, followed by Forward (9%) and Options (8%), with underlying being mostly interest rates (32%) and currencies (16%) followed in order by Equities (8%) and credit (4%)

Because of the confidential nature of the data, the structural analysis of the two networks is performed without revealing any nodes’ specific information, but rather giving a topological and statistical description of the networks. However, numbers can be given at some informative level of aggregation. The overall notional value of the sampled portfolios for instance was 78 trillion of euro on February 14, 2020, at the beginning of the turmoil caused by the COVID-19 pandemic. Figure 1 shows the percentage composition of the sampled portfolios both in terms of the type of derivative instruments and underlying asset classes. Most of the portfolios in the sample have a mixed composition along the two dimensions of interest (i.e., derivative instruments and underlying). In particular, almost 57 % of the notional can be attributed to portfolios that contain more than one type of derivative instrument, while almost 40% of the overall notional can be attributed to portfolios containing more than one type of underlying.

Being the focus on collateral networks, portfolios with no initial and variation margin requirements are not included in the sample independently from their notional size. When IMs and VMs are required instead, both ‘pure’ and ‘mixed’ portfolios are included to obtain a more comprehensive network overview of the risks in the non-centrally cleared derivative system.

## Financial network analysis

I analyze two different networks of bilateral derivative transactions which are the initial margin and the variation margin networks. The network structures to be analyzed can be encoded in the adjacency matrices. I denote with ${\varvec{A}} = (a_{ij})$ the binary adjacency matrix where $a_{ij}=1$ if there is a link between *i* and *j* (counterparties entered the contract and at least one of them posted either IMs or VMs as collateral for their net positions) and 0 otherwise. With *N* being the set of nodes and *E* being the set of edges, the network is very large being $N=36472$ and $E=64978$ on February 14, 2020, before the market turmoil caused by the COVID-19 pandemic. I consider also the weighted adjacency matrices ${\varvec{A}}^{w}_{im} = (w_{ij}^{im})$ and ${\varvec{A}}^{w}_{vm} = (w_{ij}^{vm})$ associated with the IM and VM networks, respectively, where $w_{ij}^{im}= IM_{i} + IM_{j}$ is the amount of initial margin posted by the two counterparties (gross exposure), while $w_{ij}^{vm}= VM_{j \rightarrow i}$ is the flow of money from *j* to *i*. Thus, while the initial margin network is treated as an *undirected* network (both *weighted* and *binary*), the variation margin network will be analyzed as a *weighted directed* one since it represents money flowing between institutions. In the following, I provide a structural analysis of the two networks and potential policy implications.

### The initial margin network

The network of financial derivative transactions is not fixed over time, the number of nodes and edges will depend on the period chosen for the analysis. However, as will be shown, there are general structural properties that are rather stable over time. I will particularly focus on the network structures on February 14, 2020, and March 31, 2020, thus before and after the market turmoil caused by the COVID-19 pandemic.

I begin with local network measures, in particular Fig. 1 shows the degree distribution in log–log scale before and at the end of the market turmoil. Despite the absence of central clearing through CCPs, the network of bilateral derivative exposures is characterized by a statistical abundance of financial institutions with a very large number of interconnections (i.e., degree) k with respect to the average. I thus approximate the degree distribution with a power-law behavior $f(k) \sim k^{-\alpha }$, where the fit of the coefficient is obtained by implementing the Rank-1/2 approach (Gabaix and Ibragimov 2011) which yielded a rather ‘anomalous’ exponent $\alpha < 2$, meaning the degree distribution of the bilateral binary IM networks has no finite first and second moments.

This topological feature is extremely relevant when assessing network robustness and vulnerability. Observing such a behavior for the derivative network implies robustness to random financial failures but fragility with respect to targeted attacks (Doyle et al. 2005; Gai and Kapadia 2010; Tan et al. 2015), meaning that highly interconnected financial institutions must be appropriately hedged to avoid cascade events. The derivative network is not the first real-world network showing the anomalous behavior of the tail exponent (i.e., diverging mean and variance of the degree distribution), a similar estimate has been obtained also for the worldwide airport network (Barrat et al. 2004).

**Fig. 2:**
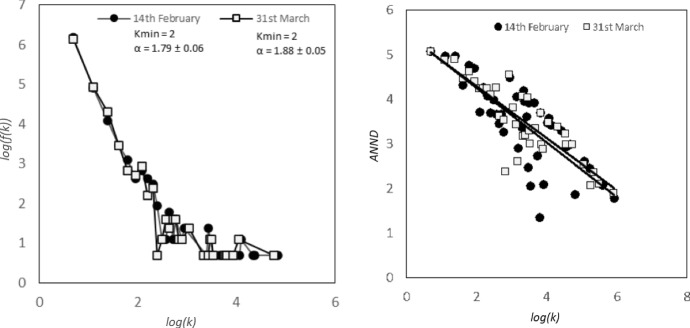
(left panel) Degree distributions of the IM network before and at the end of the COVID-19 market crash. The degree k corresponds to the number of counterparties each institution has posted initial margin to. The distributions are heavy-tailed, their functional forms have been approximated by the power-law behavior $f(k) \sim k^{-\alpha }$ with $\alpha =1.79 \pm 0.06$ before the market crash and $\alpha =1.8 \pm 0.05$. These values denote ‘anomalous’ behaviors since both the first and second moments diverge to infinite (right panel) ANND-degree scatter plot. The negative trend indicates that high-degree entities tend to be interconnected with low degree entities (i.e., negative assortativity coefficient)

**Fig. 3:**
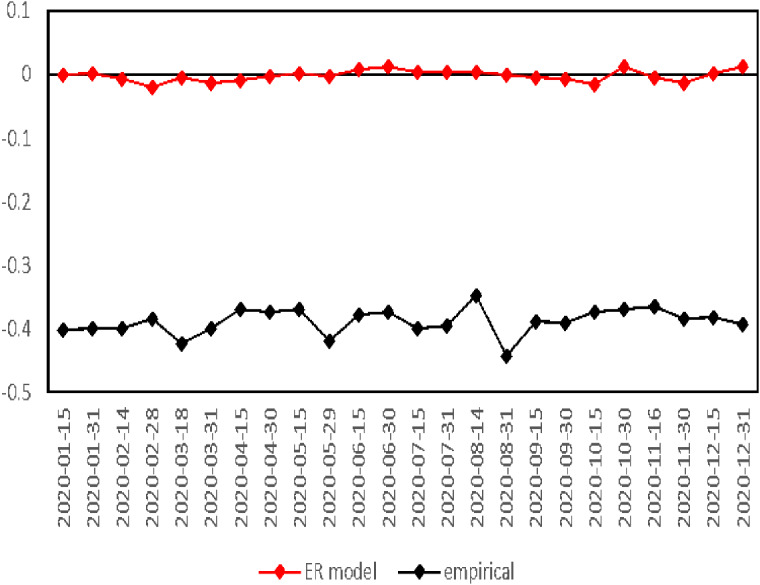
Assortativity coefficient over time for both the empirical and the Erdős–Rényi (ER) random network with the same number of nodes. The non-centrally cleared derivative market is not random at all and displays strong disassortative mixing patterns

I then proceed by exploring possible (dis)assortative patterns. I start computing the average nearest neighbor degrees (ANND) and their correlation with the degrees. The negative trend displayed in the right panel of Fig. 2 implies the network to be disassortative, since financial institutions with many interconnections tend to be mostly connected to institutions poorly interconnected. To show the temporal stability of this disassortative behavior, I calculate and plot over time the assortativity coefficient by taking daily snapshot of the network at equidistant time intervals over the period of interest. Figure 3 displays a strongly disassortative and stable dynamics for the non-centrally cleared derivative network. Moreover, the comparison with the assortativity obtained with a network having the same number of nodes but randomly generated following Erdős et al. (1960), emphasizes that a random network generation mechanism is not appropriate to describe a complex network as the one of financial derivatives. Disassortative patterns are not new in the empirics of financial networks, where interbank networks were mostly analyzed (Iori et al. 2006; Chinazzi et al. 2013; Squartini et al. 2013; Bargigli et al. 2015; Fricke and Lux 2015; Hatzopoulos et al. 2015; Iori et al. 2015; Tonzer 2015; Montagna and Lux 2017; Aldasoro and Alves 2018) and core-periphery structures were detected and modeled to explain the emergence of disassortative mixing (Lux 2015; Silva et al. 2016).

**Fig. 4:**
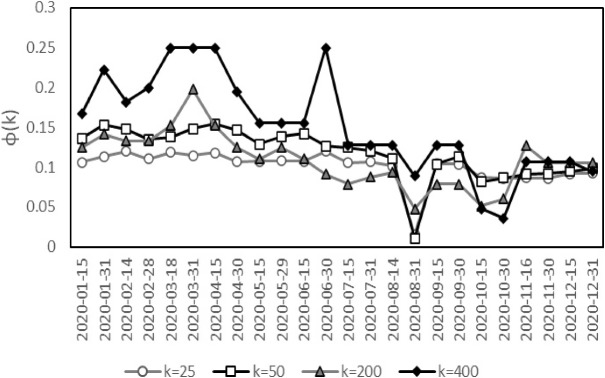
Rich-club coefficient over time. Even when considering FIs with large degrees, we do not find evidence for the presence of a core-periphery structure

Networks with a core-periphery are composed simply of two structures: the core and the periphery. Nodes in the periphery are linked only to core members, while core members are highly interconnected with each other forming cliques (complete subgraphs). Networks with a core-periphery display the so-called rich-club effect and can be heuristically quantified through the rich-club coefficient. Differently from the interbank network, the presence of a core-periphery structure to explain the disassortative mixing does not seem to fit the non-centrally cleared derivative network. While Silva et al. (2016) found the rich-club coefficient to range between 0.5 and 1 (depending on the threshold value for *k*) for the entities with several interconnections in the Brazilian interbank market, I found relatively small rich-club coefficients, ranging between 0 and 0.25 for the most interconnected financial institutions as displayed in Fig. 4.

**Fig. 5:**
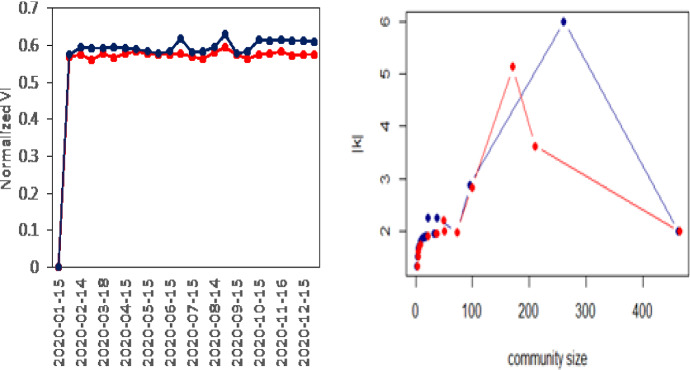
(left panel) Community structure stability through Variation of Information (normalized) over time. Comparison made for communities detected using both the modularity maximization (Louvain algorithm implementation of Blondel et al. (2008), blue color) and the Infomap approach (red color). (right panel) Average degree inside a given community versus community size (i.e., number of entities inside that community)

Given the disassortative patterns together with the presence of hubs in the network, and the absence of a core-periphery structure, it seems reasonable to believe each financial institution acting as a hub to create its own community in a hierarchical network where all peripheral members (inside the community) connect only to community hubs. The procedure implemented to partition the network in communities is the modularity maximization approach (Clauset et al. 2004; Newman 2006)^5^. The goal of modularity maximization is the uncovering of communities of nodes whose internal interactions are stronger compared to the inter-community ones, and maximally unexplained by a null model taken as benchmark which is the configuration model (CM). To check the robustness of the detected communities (i.e., ensuring the partitioning does not heavily depend on the methodological approach chosen) I follow also the Infomap approach of Rosvall and Bergstrom (2008) and compare the results. I then check the temporal stability of the detected partitions by computing an information-theoretic measure known as *variation of information* (VI) which can be used to quantify the difference between two partitions (Meilă 2007). Given two partitions of the nodes at two different points in time, $\gamma ^{t}$ and $\gamma ^{t+\delta t}$, the VI is defined as

1 $$\begin{aligned} \begin{aligned} VI(\gamma ^{t},\gamma ^{t+\delta t})&= [H(\gamma ^{t}) - I(\gamma ^{t},\gamma ^{t+\delta t})] + [H(\gamma ^{t+\delta t}) - I(\gamma ^{t},\gamma ^{t+\delta t})]\\&= H(\gamma ^{t} \mid \gamma ^{t+\delta t}) + H(\gamma ^{t+\delta t} \mid \gamma ^{t}) \end{aligned} \end{aligned}$$

where $I(\gamma ^{t},\gamma ^{t+\delta t})$ is the *mutual information* between the two partitions while $H(\gamma ^{t})$ and $H(\gamma ^{t+\delta t})$ are their *entropies*. These terms can be rearranged in the two conditional entropies which measure, respectively, the amount of information lost about $\gamma ^{t}$ and the one that must be gained about $\gamma ^{t+\delta t}$ when moving from $\gamma ^{t}$ to $\gamma ^{t+\delta t}$. The VI measure possesses some desirable properties: it satisfies all the metric axioms (i.e., it is a real distance) and is bounded from above by $\ln N$ to control for the magnitude of the variation among clusters, making possible to compare clustering across datasets and across algorithms as well.^6^ Both approaches reveal a dynamic structure with around 60% change in communities month by month, meaning many nodes change each month the community to which they belong. This behavior is confirmed across different community detection methodologies as shown in Fig. 5. In the right panel of the same Fig. 1 also plots the average degree |*k*| computed inside a given community versus the size of the latter comparing again the results across the two different community detection approaches. Results are again quite consistent: the two approaches manage to identify almost perfectly the smaller communities and the largest one, with the only difference being that the modularity approach clusters together a group of nodes in one large community (the second one in terms of size), while the Infomap approach splits the same large group into two distinct communities. Apart from this discrepancy in the size and associated degrees of two communities, the rest of the clusters closely overlap suggesting the presence of dynamic community structures over time.

**Fig. 6:**
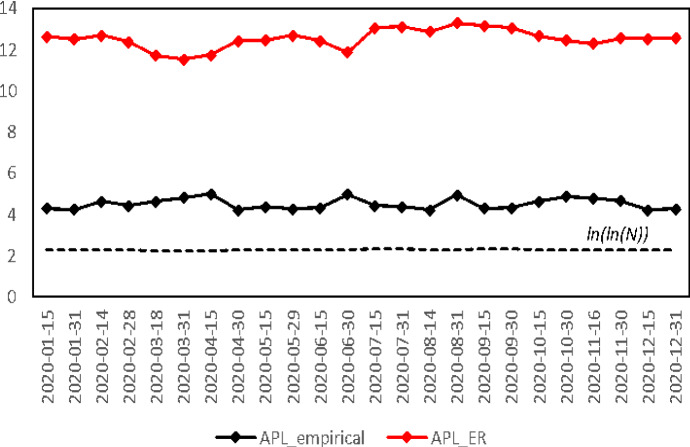
Average Path Length over time for both the empirical and the ER random network with the same number of nodes. Random networks are capable to generate small APLs, which is a key feature of the ‘small-world phenomenon.’ The derivative network displays a very short APL which closely tracks the logarithm of N, pointing in favor of an ‘ultra-small’ world

I finally look at the average path length (APL). Many real-world networks exhibit the so-called small-world property. Being ‘small-world’ means in technical terms to observe a network diameter $D \sim \ln N$ (Fronczak et al. 2004), meaning the shortest distance between the two most distant nodes increases very slowly with the number of nodes. However, the derivative networks in the non-centrally cleared space in this sense are very small, being the APL $\sim \ln (\ln N)$ and pointing in favor of an ‘ultra-small world’ phenomenon (Cohen and Havlin 2003) as typically observed in scale-free networks (Barabási and Albert 1999). In Fig. 6, the empirical APL of the derivative network is computed and plotted over time. To give an idea of the magnitude of the ultra-small world effect, the empirical APL is compared with the one we would observe, keeping the same number of nodes at each point in time, in a random network generated following (Erdős et al. 1960).

The presence of an ultra-small derivative market even without central clearing is particularly relevant for policymakers and could act as a double-edged sword: Adverse shocks can propagate in the network affecting many institutions very quickly, at the same time being the network ultra-small is much faster to identify many critical channels of contagion. The first typical and smart approach is the surveillance of highly interconnected and large institutions. Still, understanding which channels should be monitored starting from these nodes can be very difficult. In what follows, the adoption of spanning trees will be proposed as filtering tools to select the channels linking all the institutions in the fastest way and with the maximum level of exposure. The tool might be complementary to other well-established supervision approaches adopted by policymakers.

#### Identifying the maximum exposure channels: the maximum spanning tree approach

Since the seminal work of Mantegna (1999) on the hierarchical structures of financial markets, Minimum Spanning Trees (MST) have been mainly used in the literature to filter out relevant information from stock markets for both asset allocation purposes (Onnela et al. 2003; Tumminello et al. 2010; Peralta and Zareei 2016; Raffinot 2017) and the structural analysis of financial market dynamics during the COVID-19 global pandemic (Zhang et al. 2020). Given the complexity of derivative transactions and their relevance from a financial stability perspective, I propose in this work the adoption of Spanning Trees to filter out a subgraph containing the highest derivative exposures in the non-centrally cleared derivative network.

##### Definition 1

(*Spanning Tree*) Given a graph $G = (N, E)$, with *N* being the set of nodes and *E* being the set of edges, a Spanning Tree is a connected acyclic undirected subgraph of G connecting all the nodes in G.

##### Definition 2

(*Maximum Spanning Tree*) Given a weighted connected graph $G = (N, E, W)$, with *N* being the set of nodes, *E* being the set of edges, and *W* being the set of weights, a Maximum Spanning Tree (Max-ST) is a spanning tree T of G maximizing $\sum _{e \in T}W(e)$.

The approach is conceptually identical to the MST with the crucial difference that I am going to maximize the weight of the path which goes through all the financial institutions, leading then to the Maximum Spanning Tree (Max-ST). Being the general weight of an edge $w_{ij}^{im}= IM_{i} + IM_{j}$, the Max-ST then contains a subgraph characterized by N nodes and N-1 edges for which the amount of initial margins is maximized. This is crucial from a policy perspective since the biggest exposures in terms of potential losses are those to be closely tracked and supervised. This filtering procedure can be very useful from a macroprudential perspective. When comes to assessing financial stability is fundamental to both focus on sizeable exposures and act in a timely manner to avoid contagion in the financial system. In complex networks characterized by large amount of transactions as in the financial derivative markets, it is very hard to keep the focus on the entire system under control without losing crucial information on some of its entities. To this end, the Max-ST network possesses two interesting properties. First, all the financial institutions are connected in the tree with the minimum amount of links needed, meaning that is always possible to find a path on the tree to reach one financial institution starting from another one. This allows the supervisors to focus on a small subset of transactions, instead of tracking all of them, without removing any financial institution from the sample. Second, the path directly (i.e., no cycles) connecting two generic institutions on the tree has the maximum possible weight. This implies that given a default propagation chain of length $L \le N-1$, the chain of length *L* with the maximum potential loss in the derivative system is always on the Max-ST.

It is worth stressing that only direct contagion paths are located on the tree, with no loops in the potential chain of defaults. When the distress is not promptly mitigated, and feedback effects through cycles arise in the default propagation mechanism, the final loss in the system might be even underestimated with a spanning tree approach. Nevertheless, whenever the length of the chain through which the default propagates in the system remains bounded by $N-1$, the Max-ST displays the path on which the loss would be maximal given that length. This might represent crucial information for supervising authorities, especially when there is no evidence prior about the specific path on the network to be supervised starting from a specific entity, to timely act when the propagation starts.

**Fig. 7:**
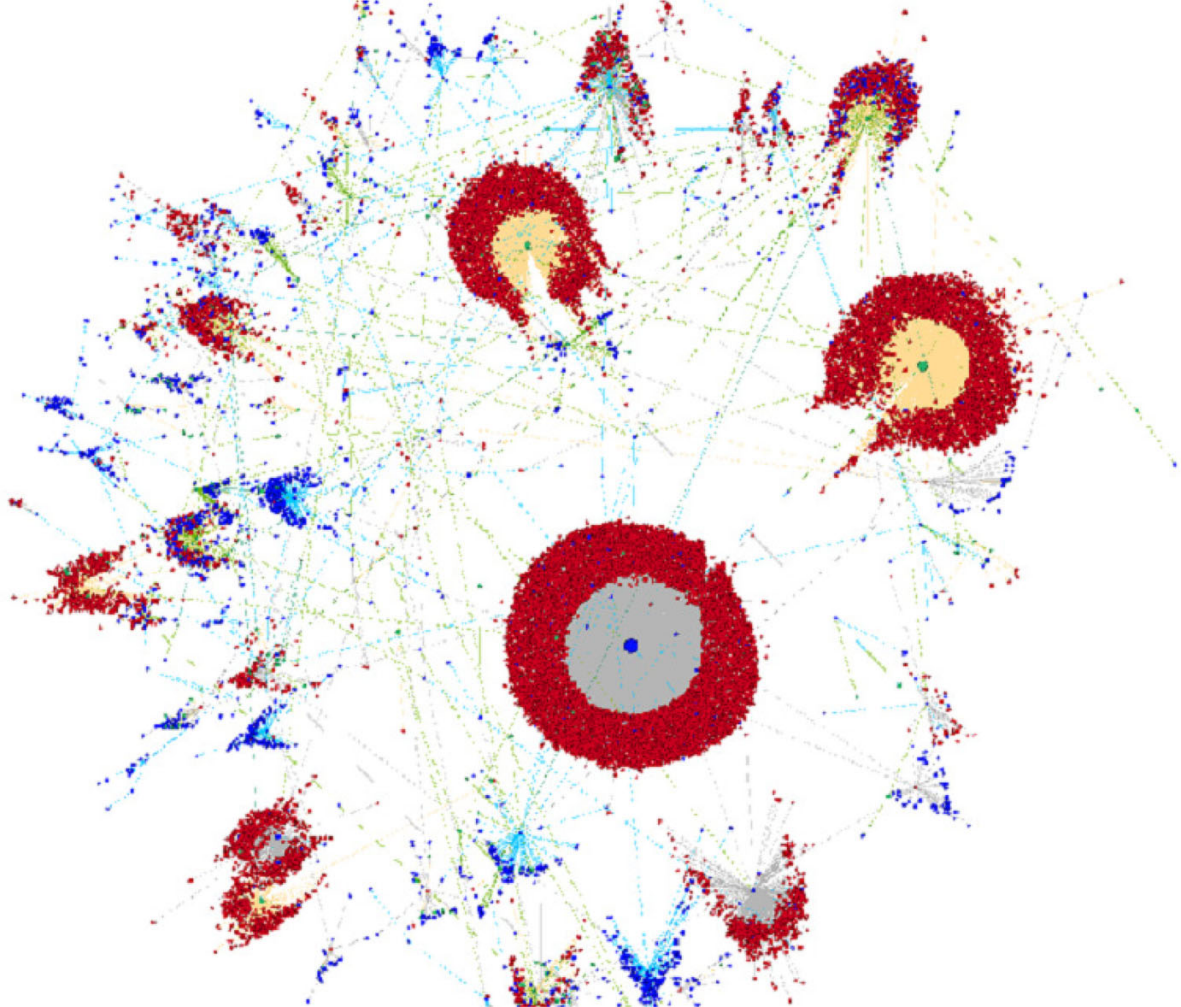
Graphical representation of the Max-ST of the initial margin network. Nodes in blue represent companies in the EA, in green European Union (EU) but not EA, and in red companies outside the EU. The edges in gray link EA with extra-EU nodes, yellow edges connect EU (but not EA) to extra-EU nodes, green edges connect EU to EU nodes, while light blue connect EA to EA nodes

**Fig. 8:**
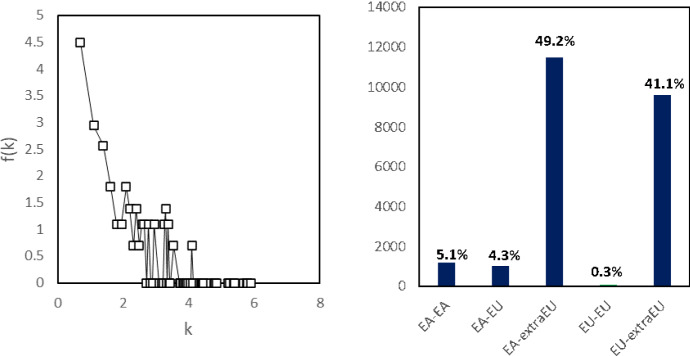
Degree frequency of the Maximum Spanning Tree on a log–log scale (left) and jurisdictions in which the largest exposure takes place. Around 90% of the exposures laying on the Max-ST represent cross-border transactions between EA/EU and extra-EU counterparties

The graphical representation of the Max-ST is provided in Fig. 7, while in Fig. 8 the degree distribution is shown together with additional information on the jurisdictions involved in the transactions. As expected, the same fat-tail behavior is observed for the entire IM network. More interestingly, around 90% of the exposures on the maximum spanning tree represent transactions with counterparties outside the EA. Being the greatest exposure across jurisdictions, strict cooperation among authorities and policymakers is highly needed to implement effective supervision practices for global financial stability purposes. I now move to the analysis of the directed variation margin network, thus focusing on the liquidity flows faced by the nodes in the network during the COVID-19 market turmoil.

### The variation margin network

Variation margins are exchanged in cash at least daily to reflect mark-to-market changes on counterparties’ positions caused by price changes on the underlying of the derivative instrument. They represent flows of money moving from one counterparty to another, which can be at the origin of liquidity stress for counterparties. As for the initial margin network, I collect variation margin flows over the period February 14–March 31, 2020, in order to include the market crash caused by the beginning of the COVID-19 pandemic. Recalling the weighted adjacency matrix to be $A^{w}_{vm} = (w_{ij}^{vm})$ with $w_{ij}^{vm}= VM_{j \rightarrow i}$ the flows from *j* to *i*, I focus on the out-strength and in-strength distributions to capture outflows and inflows faced by institutions. I also compute the overall strength for each node so to identify losers and gainers, over the period of interest (i.e., overall positive strength simply means the inflows were larger than the outflows and vice versa), showing their distributions as well. The out/in-strength measure for a generic node *i* then read simply as

2 $$\begin{aligned} S_{i}^{\textrm{Out}}= & {} \sum _{j}w_{ji}^{vm} \end{aligned}$$

3 $$\begin{aligned} S_{i}^{\textrm{In}}= & {} \sum _{j}w_{ij}^{vm} \end{aligned}$$

while the overall strength (net-inflows/outflows) by consequence read as $S_{i} = S_{i}^{\textrm{In}} - S_{i}^{\textrm{Out}}$. All these strength measures tell us how the wealth at stake via bilateral derivative transactions has been distributed among transacting entities during the COVID-19 market turmoil.

**Fig. 9:**
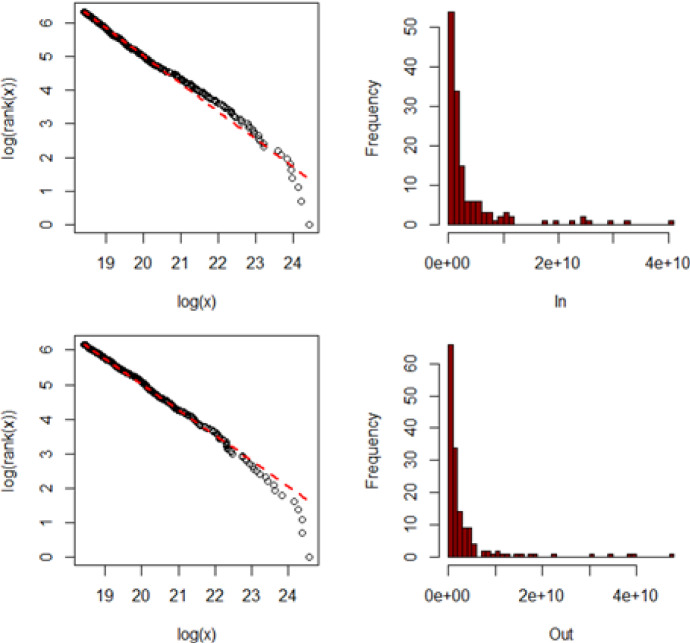
In-strength and out-strength distributions on a log–log scale (left panel) and empirical frequency distributions (right panel)

Variation margins can be exchanged even when no initial margin is required to enter the derivative contracts, which typically happens for very small exposures among counterparties. This makes the variation margin network very dense and full of small transactions almost negligible from a financial stability perspective and which would spike the distribution over zero. I then reconstruct the network setting a relatively low edge threshold $\tau = 10$ million, removing all of those ‘small’ positions that generated less than 10 million of cumulative inflows/outflows over the analyzed period. Figure 9 then displays the in-strength and out-strength distributions, respectively, from which it is possible to observe the fat-tail behavior typically observed in many real-world networks. These distributions conceptually are wealth distributions where a small fraction of entities account for a very large fraction of all the flows in the system. I then model the distributions as Pareto type I distributions which can be written as

4 $$\begin{aligned} F(x) = 1 - \left( \frac{x_{\min }}{x} \right) ^{\alpha }, \;\;\;\;\; x_{\min }> 0, \alpha > 0 \end{aligned}$$

The maximum likelihood estimator (MLE) of $x_{\min }$ is straightforward, since it is the minimum of the sample. Given the known $x_{\min }=\tau$, I estimate the MLE ${\hat{\alpha }}$ of $\alpha$. Note that the MLE of $\alpha$ is biased but consistent. Moreover, given the size of the network the bias is entirely negligible and does not affect the results. The estimates yielded ${\hat{\alpha }}=0.6369$ and ${\hat{\alpha }}=0.6563$ for the in-strength and out-strength distributions, respectively. The estimates imply both the first and second moments of the distributions to diverge to infinite asymptotically with the number of entities transacting in derivative markets. This brings tremendously important policy implications. Diverging moments for the out-strength distribution imply the presence of extreme outflows worsening liquidity stress during market crashes and thus calling for central banks’ interventions. I then look at the overall strength distributions, splitting the sample between those entities which lost ad those which earned money over the period of interest. For these net-wealth distributions estimates yielded ${\hat{\alpha }}=0.6697$ and ${\hat{\alpha }}=0.7036$ for the net-inflows and net-outflows respectively, signaling extreme earnings and losses behavior in non-centrally cleared derivative markets during the COVID-19 market crash.

Since the results might depend on the threshold $\tau =10$ million, it is interesting to check the sensitivity of the results with respect to $\tau$. These estimates on real data could act as a reference for future network analyses on derivative markets, since bilateral transactions data are not publicly available and have to be simulated starting from aggregate data. I then reconstruct the network using different thresholds and I estimate again $\alpha$ for each threshold adopted. The results in Table 1 show how both the size of the network (in terms of edges and nodes) and the parameter’s estimates change when different threshold values are used. It is possible to see that the estimates of the scaling coefficients are sensitive to the choice of the threshold adopted, still they tend to be $\le 1$. These results, obtained for very different values of the threshold after which the power-law distribution is fitted, confirm the very high degree of heterogeneity in the derivative exposures and the existence of extreme transactions with respect to the average.

**Table 1:** Estimates table for the Pareto type I’s parameter $\alpha$ for different network structures induced by the threshold chosen

$\varvec{{\hat{\alpha }}}$	In-strength	Out-strength	Net-inflows	Net-outflows
$\tau =1$ million	0.4322	0.4596	0.4456	0.4841
$$N=5652$$				
$$E=26276$$				
$\tau =10$ million	0.6369	0.6563	0.6697	0.7036
$$N=2351$$				
$$E=8782$$				
$\tau =100$ million	0.8291	0.7378	0.9722	0.8154
$$N=561$$				
$$E=1349$$				
$\tau =500$ million	0.8341	0.7674	1.122	1.092
$$N=144$$				
$$E=167$$				

Always diverging second moment, almost always non-finite mean

It is worth mentioning that institutions involved in non-centrally cleared transactions might be transacting at the same time in the centrally cleared space as well. Then, one could argue that extreme counterparties’ positioning and related net-outflows in the non-centrally cleared space might be there to simply hedge the exposures in the centrally cleared one or vice versa. In this case, those net-outflows would not cause liquidity stress since they would be netted by the net-inflows stemming from other exposures with different positioning strategies in the centrally cleared space. For this reason, for each of the sampled institutions acting in the non-centrally cleared space, I control for the presence of net-inflows stemming from the centrally cleared. I thus compute the following quantity.^7^

$$\begin{aligned} \mathrm S^{\textrm{hedged}}_{i} = \textrm{Out}^{\textrm{ncc}}_{i} - In^{\textrm{cc}}_{i} \end{aligned}$$

**Fig. 10:**
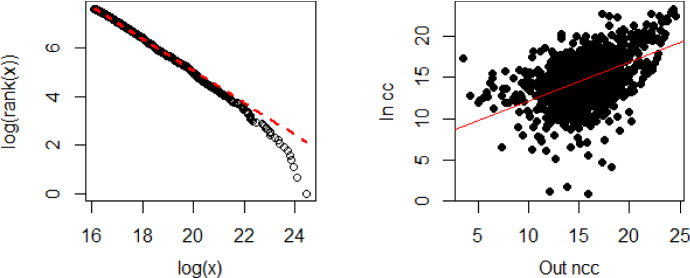
$S_{\textrm{hedged}}$ distribution in log–log scale on the left hand side, with its two components $\textrm{Out}_{\textrm{ncc}}$ and $In_{\textrm{cc}}$ on a scatter plot on the right hand side

Figure 10 displays the distribution of $S^{\textrm{hedged}}$ on a log–log scale, together with a scatter plot showing for each outflows in the non-centrally cleared space the associated inflows in the centrally cleared one. As expected, hedging activity is in place to some extent since outflows and inflows in the two spaces tend to be positively correlated. For some counterparties, inflows completely offset outflows. Still, hedging between the two spaces is only partial and many counterparties experience large net-outflows. Two main facts can be graphically appreciated in Fig. 10 indeed. First, inflows from centrally cleared exposures cover outflows in the non-centrally cleared space only partially. Second, even when offsetting outflows with inflows across the two spaces, extreme outflows appearing in the right tail of the distribution are detected. Moreover, the ML estimate of the Pareto type I coefficient yielded results implying diverging first and second moments as well.

For a complete and proper assessment of risk stemming from derivative exposures, it must be kept in mind that multiple hedging activities can be implemented by counterparties. One simple example would be the one in which a counterparty enters not only in derivative contracts (either cleared or not) with a long/short position but also in the underlying of the derivative but with opposite positioning. This would require the access, for each counterparty, to each financial asset hold in their balance sheet and goes well beyond the scope and the possibilities of this work. Keeping in mind all of these warnings, this paper uncover for the first time a huge part of the non-centrally cleared derivative markets, shedding lights on its structure and capability to generate extreme positions and liquidity outflows which are not offset in the centrally cleared space for many counterparties. This leads us to the conclusions.

## Conclusion

After the 2008 global financial crises, regulators made tremendous efforts to make derivative markets safer by requiring the adoption of risk mitigation techniques and the introduction of central clearing obligations to reduce counterparty credit risk. Despite the evident improvements, derivative instruments still embed substantial risks, especially when used in opaque ways as shown by the default of Archegos in March 2021. Non-centrally cleared markets constitute a substantial share of the overall derivative markets, but there is either little or no focus at all on these bilateral markets (possibly because of no access to such confidential data) in the current research literature which mainly focused on central clearing instead. This paper shed light on the network structure of non-centrally cleared derivative markets, providing a detailed analysis of risks and liquidity dynamics under stressed periods.The non-centrally cleared network of derivative transactions is found to display very fat-tails on degree and strength distributions even without the presence of central clearing counterparties, with anomalous exponents signaling the presence of extreme positions causing large liquidity outflows in stressed periods. Being the network ‘ultra-small,’ acting in a timely in case of extreme losses is fundamental for policymakers to avoid contagion. Moreover, the highest exposures are across jurisdictions, calling for cooperation among different authorities.

## Supplementary information

The data supporting the research findings are highly confidential and cannot be made available publicly. The papers have been cleared for external submission to journals by the European Central Bank. Codes and anonymized statistical outputs can be shared and are available upon request.

## Appendix A: Network metrics definitions

*Average nearest neighbor degrees (ANND)*. Being $A_{i}$ the *i*-th row of the adjacency matrix A the ANND for node *i* is computed as

$$\begin{aligned} \textrm{ANND}_{i} = \frac{{\varvec{A}}_{(i)}\varvec{A1}}{{\varvec{A}}_{(i)}{\varvec{1}}} \end{aligned}$$

and tell us what is, on average, the degree of the neighbors of node *i*.

*Assortativity coefficient*. It is the Pearson correlation coefficient of the degrees $k_{i}$, $k_{j}$ at either ends of an edge $e_{ij}$ (Newman 2002), that is

$$\begin{aligned} \rho = \frac{1}{\sigma _{f}^{2}}\sum _{e_{ij}}k_{i}k_{j}(f_{(k_{i}k_{j})} - f_{k_{i}}f_{k_{j}}) \end{aligned}$$

where $f_{k_{i}}$ denotes the distribution of the remaining degree (i.e., total degree of a node on randomly chosen edge minus one), $f_{(k_{i}k_{j})}$ denotes the joint distribution of the remaining degrees of the two nodes at either ends of the randomly chosen edge, $\sigma _{f}^{2}$ is the empirical variance of the degree distribution to normalize the coefficient.

*Rich-club coefficient*. Being $N_{>k}$ the number of nodes having degree higher than a given threshold *k* and $E_{k}$ the number of edges among those nodes, the rich-club coefficient is computed as (Zhou and Mondragón 2004)

$$\begin{aligned} \phi (k) = \frac{2E_{k}}{N_{>k}(N_{>k}-1)} \end{aligned}$$

where $N_{>k}(N_{>k}-1)$ is the maximum number of possible edges which could exist among those $N_{>k}$ nodes.

*Average path length (APL)*. It is the average of all the shortest paths connecting any possible pairs of nodes in the network. When the graph is binary, it becomes by construction the number of steps to be done on average along these shortest paths connecting any pairs of nodes. Thus, being $d_{(i,j)}$ the shortest distance between two generic nodes i and j, we have $APL = \sum _{i \ne j}d_{(i,j)}/N(N-1)$ where $N(N-1)$ is the total number of paths.

*Modularity*. The modularity $Q(\gamma )$ of a partition $\gamma$ of the *N* nodes of a network is defined as

$$\begin{aligned} Q(\gamma ) = \frac{1}{2m}\sum _{ij}(a_{ij} - E[a_{ij}])\delta (\gamma _{i},\gamma _{j}) \end{aligned}$$

where $2m = \sum _{ij}a_{ij}$ is the total edge weight in the network while $E(a_{ij})$ is the expected value of the entry of the adjacency matrix under a null model. The null model is the configuration model (CM) which randomizes the network topology still preserving the empirical degree sequence. It reads as $E(a_{ij}) = k_{i}k_{j}/2\,m$ and represents the probability for *i* and *j* to be randomly connected given their degree sequence assuming a uniform probability distribution over the edges. The Kronecker delta function $\delta (\gamma _{i},\gamma _{j})$ controls that only nodes belonging to the same community contribute to the increase in modularity when maximizing $Q(\gamma )$. The partition $\gamma$ maximizing $Q(\gamma )$ is the one having community of nodes whose internal interactions are maximally stronger that the ones they would have under the null model taken as a benchmark.

*Community tracking and the IV metric*. Consider two partitions of the nodes into mutually disjoint subsets, obtained through any suitable clustering method at two different points in time, $\gamma ^{t} = \{\gamma _{1}^{t}, \gamma _{2}^{t},..., \gamma _{K}^{t} \}$ and $\gamma ^{t+\Delta t} = \{\gamma _{1}^{t+\Delta t}, \gamma _{2}^{t+\Delta t},..., \gamma _{K}^{t+\Delta t} \}$. I assume each node has the same probability of being picked, the probabilities of a generic node being, respectively, in the clusters $\gamma _{k}^{t}$ and $\gamma _{k'}^{t+\Delta t}$ are $P(k) = n_{k}/N$ and $P(k') = n_{k'}/N$. I then follow Meilă (2007) quantifying the entity associated with the two partitions by computing their entropies

$$\begin{aligned} H(\gamma ^{t}) = - \sum _{k=1}^{K} P(k) \ln P(k) \\ H(\gamma ^{t}) = - \sum _{k'=1}^{K'} P(k') \ln P(k') \end{aligned}$$

which is always nonnegative and it is equal to zero when there is only one large cluster containing all the nodes (i.e., no uncertainty). I quantify the information that one partition has about the other by computing the *mutual information* as follows:

$$\begin{aligned} I(\gamma ^{t}, \gamma ^{t+ \Delta t}) = \sum _{k=1}^{K}\sum _{k'=1}^{K'} P(k,k') \ln \frac{P(k,k')}{P(k)P(k')}. \end{aligned}$$

The VI metric computed as

$$\begin{aligned} \begin{aligned} VI(\gamma ^{t},\gamma ^{t+\delta t})&= [H(\gamma ^{t}) - I(\gamma ^{t},\gamma ^{t+\delta t})] + [H(\gamma ^{t+\delta t}) - I(\gamma ^{t},\gamma ^{t+\delta t})]\\&= H(\gamma ^{t}\mid \gamma ^{t+\delta t}) + H(\gamma ^{t+\delta t} \mid \gamma ^{t}) \end{aligned} \end{aligned}$$

represent the amount of information that we lose plus the one we must gain when movingfrom clustering $\gamma ^{t}$ to clustering $\gamma ^{t+\Delta t}$. The higher the metric, the lower the stability of the detected communities. The VI can be normalized by using its upper bound which is the $\ln N$, so $NVI = VI/ \ln N$.

## Appendix B: Pareto-type I estimation details

I estimate the coefficient $\alpha$ of $F(x) = 1 - (x_{\min }/x)^{\alpha }$ by maximum likelihood. Assuming $X_{1},..., X_{N}$ to be independent and identically Pareto distributed random variables, the MLE of $\alpha$ can be easily derived and is equal to

$$\begin{aligned} {\hat{\alpha }} = N/\sum _{i=1}^N \ln \frac{X_{i}}{x_{\min }}. \end{aligned}$$

When $\alpha < 1$ as in the VM networks analyzed, both firsts and second moments of outflows and inflows diverge to infinite since

$$\begin{aligned} E(X) = \int _{x_{\min }}^{\infty } x \frac{\alpha (x_{\min })^{\alpha }}{x^{\alpha + 1}} = \alpha (x_{\min })^{\alpha } \int _{x_{\min }}^{\infty } 1/x_{\alpha } = \alpha (x_{\min })^{\alpha } \left[ \frac{x^{1-\alpha }}{1-\alpha } \right] ^{\infty }_{x_{\min }} = \infty \end{aligned}$$

which signals the presence of extreme liquidity outflows and inflows on derivative positions in non-centrally cleared markets. Analogous computations follow for the second moment as well. As shown in Rytgaard (1990), the MLE of $\alpha$ is biased but consistent, since $E({\hat{\alpha }}) = \alpha N / (N-1)$.

## Appendix C: Details on data collection and cleaning

*Dealing with misreporting.* Each trade must be reported in EMIR by both counterparties. As illustrated in the data description part, both initial and variation margins do not refer to a trade standalone between counterparties, but they rather refer to their overall bilateral portfolios (i.e., net positions resulting from multiple trades). An illustrative example which makes use of a pseudo-EMIR table appearing in EMIR is shown in Table 2. Since margins are the same for different Trade id whenever they belong to the same portfolio, we must sample margins only once for each portfolio. However, two reporting errors might appear in the data posing challenges for network reconstruction:

**Table 2:** Pseudo-EMIR table showing different trades belonging to the same portfolio and thus containing the same amount of margin posted and received by the two counterparties

Trade	Portfolio	Reporting	Other	Contract	Asset	Notional	IM(VM)	IM(VM)
Id	code	cpty	cpty	type	class		posted	received
01	AB	A	B	Options	Equities	1000	50	10
02	AB	A	B	Futures	Equities	2000	50	10

The case of a mixed portfolio in terms of derivative instruments is illustrated

**Table 3:** Pseudo-table showing the situation in which different margins are reported for trades belonging to the same portfolio

Trade	Portfolio	Reporting	Other	Contract	Asset	Notional	IM(VM)	IM(VM)
Id	code	cpty	cpty	type	class		posted	received
01	AB	A	B	Options	Equities	1000	**50**	10
02	AB	A	B	Futures	Equities	2000	49	**11**

Typically, this happens for reporting mistakes and values are very close to each other. When this happen, always the maximum amount of margin reported is taken (values in bold)

**Table 4:** Pseudo-table showing disagreement in the reporting of the two counterparties

Portfolio	Reporting	Other	Notional	IM(VM)	IM(VM)
code	cpty	cpty		posted	received
AB	A	B	3000	**50**	10
AB	B	A	3000	**10**	40

In particular, what A reported as ’posted to B’ in the first row (i.e., 50) is not consistent with what B reported as ’received from A’ in the second row (i.e., 40). When situations like this happens, I fix the posted fields (values in bold) as the default options

1. *Misreporting of the margins across different Trade id*. When different trades (identified by a trade id) belong to the same portfolio, the margin field should be equal across trades as shown in the pseudo-EMIR Table 2. Still, it can happen to observe different numbers. In these situations, I always take the maximum amount of margin reported by the counterparty as shown in Table 3. This misreporting issue is not particularly challenging because even if margins sometimes differ across trades id, they are typically very close to each other.
2. *Counterparties report inconsistent data*. This issue is in principle more challenging. Since EMIR is a double-reporting based system, the two trade records reported by two generic counterparties involved in the trade of interest should be, ideally, consistent. Unfortunately, this is not always the case. Sometimes it happens the situation in which the amount of margins that A said to have posted to be B for instance, is different from the amount of margins that B said to have received from A as shown in Table 4. Whenever these inconsistencies between ’posted’ and ’received’ fields arise, a rule to select a data field is needed. I always choose the posted as the default option. Fortunately, these discrepancies are not dramatically big and do not alter the messages conveyed in the paper. Then, the final table with consistent data will be the one displayed in Table 5.

**Table 5:** Final and cleaned pseudo-table on which the network reconstruction and related analyses take place

Portfolio	Reporting	Other	Notional	IM(VM)	IM(VM)
code	cpty	cpty		posted	received
AB	A	B	3000	50	10
AB	B	A	3000	10	50

The ‘received’ field in the second row which was equal to 40, has been replaced with the posted field (equal to 50) in the first row

## Footnotes

1 Statistical release from the BIS website available at Statistical release: OTC derivatives statistics at end December 2021.

2 The statement is the result of internal computations undertaken by the author on confidential EMIR data and is *limited* to the jurisdictions overviewed by the ECB.

3 Precisely, variation margins were simulated from a zero-mean Normal distribution. As it will be shown in the remainder of this paper, variation margins are all but normally distributed and zero-mean random variables, unfortunately. As a consequence, liquidity stress stemming from derivative positions could be strongly underestimated under the Normality assumption.

4 I refer interested readers to the Appendix for more details on the data collection process.

5 See Appendix A for details on the modularity optimization approaches. All the codes are publicly available in the igraph R package.

6 For more details, I refer the interested readers to Meilă (2007) and to Appendix A.

7 Out refers to net-outflows and In refers to net-inflows. From now on, I will simply refer to outflows and inflows across the two spaces, taking for granted that flows have been netted inside each space.
